# Surface-anchored microbial enzyme-responsive solid lipid nanoparticles enabling colonic budesonide release for ulcerative colitis treatment

**DOI:** 10.1186/s12951-023-01889-0

**Published:** 2023-05-02

**Authors:** Yipeng Zhang, Liying Wang, Zi-Dan Wang, Quan Zhou, Xuefei Zhou, Tianhua Zhou, Yi-Xin Guan, Xiangrui Liu

**Affiliations:** 1 Department of Pharmacology and Department of Radiology of Sir Run Run Shaw Hospital, Zhejiang University School of Medicine, Hangzhou, 310016 China; 2 Zhejiang Key Laboratory of Smart Biomaterials, College of Chemical and Biological Engineering, Zhejiang University, Hangzhou, 310027 China; 3 Innovation Center of Yangtze River Delta, Zhejiang University, Jiaxing, 314100 China; 4 Department of Cell Biology, Zhejiang University School of Medicine, Hangzhou, 310058 China; 5 International Institutes of Medicine, The Fourth Affiliated Hospital of Zhejiang University School of Medicine, Yiwu, 322000 China; 6 Cancer Center, Zhejiang University, Hangzhou, 310058 China; 7 Department of General Surgery, Sir Run Run Shaw Hospital, Zhejiang University School of Medicine, Hangzhou, 310016 China

**Keywords:** Solid lipid nanoparticles, Ulcerative colitis, Microbial enzyme, Colon-targeted oral drug delivery system, Sodium cellulose sulfate, Budesonide

## Abstract

Colon-targeted oral drug delivery systems (CDDSs) are desirable for the treatment of ulcerative colitis (UC), which is a disease with high relapse and remission rates associated with immune system inflammation and dysregulation localized within the lining of the large bowel. However, the success of current available approaches used for colon-targeted therapy is limited. Budesonide (BUD) is a corticosteroid drug, and its rectal and oral formulations are used to treat UC, but the inconvenience of rectal administration and the systemic toxicity of oral administration restrict its long-term use. In this study, we designed and prepared colon-targeted solid lipid nanoparticles (SLNs) encapsulating BUD to treat UC by oral administration. A negatively charged surfactant (NaCS-C12) was synthesized to anchor cellulase-responsive layers consisting of polyelectrolyte complexes (PECs) formed by negatively charged NaCS and cationic chitosan onto the SLNs. The release rate and colon-specific release behavior of BUD could be easily modified by regulating the number of coated layers. We found that the two-layer BUD-loaded SLNs (SLN-BUD-2L) with a nanoscale particle size and negative zeta potential showed the designed colon-specific drug release profile in response to localized high cellulase activity. In addition, SLN-BUD-2L exhibited excellent anti-inflammatory activity in a dextran sulfate sodium (DSS)-induced colitis mouse model, suggesting its potential anti-UC applications.

## Introduction

Ulcerative colitis (UC) is a disease with high relapse and remission rates associated with immune system inflammation and dysregulation localized within the lining of the rectum and colon [1]. Although UC is not a fatal disease, the symptoms of UC generally worsen over time and develop from mild to severe. Current available medications cannot completely cure the disease, and first-line treatments aim to relieve the symptoms and achieve clinical remission [2, 3] and involve the use of anti-inflammatory steroids [2–4]. Steroids have long been used to induce remission in UC patients. According to the guidelines of the European Crohn’s and Colitis Organization (ECCO), topically applied steroids offer advantages over systemic steroids, including localized and targeted treatment with fewer systemic side effects [5–8]. However, some patients have difficulty accepting topical treatments because of the inconvenience of the administration route. In addition, the rectal administration of foam and enema formulations mainly results in drug delivery to the rectum and distal colon [9, 10], limiting the application scope. In contrast, oral administration therapy requires higher doses of drugs with low oral bioavailability and high systemic toxicities.

Oral drug delivery is one of the most convenient and common drug administration routes. Colon-targeted oral drug delivery systems (CDDSs) are desirable for UC treatment [11], since the major inflammation location is the colon. Endogenous gastrointestinal (GI) microbial flora plays a key role in maintaining homeostasis, and there are significant variations in the bacterial diversity and microbial communities in the human intestine [12]. The breakdown of dietary fibers by the cellulolytic bacteria in the large intestine, mostly the *Fibrobacter* and *Ruminococcus* genera, critically contributes to improving nutrition and health in both herbivorous and omnivorous mammals, including humans [13]. Importantly, the tremendous difference between the density of cellulolytic bacteria in the colon (0.5 × 10^8^-12.2 × 10^8^/g) and that in the small intestine (10^3^/g) results in a sharp gradient of cellulase-enzyme activity, providing a specific stimulus for CDDSs based on cellulose derivatives [14]. Sodium cellulose sulfate (NaCS) is a cellulose derivative made from sulfonated natural cellulose with good water solubility, biocompatibility and biodegradability [15]. Polyelectrolyte complexes (PECs) formed by negatively charged NaCS and cationic chitosan can be used as microbial cellulase-responsive materials to prepare colon-specific degradable capsules [16]. However, *in vivo* evaluations of NaCS-based colon-targeted materials are still lacking.

Solid lipid nanoparticles (SLNs) have emerged as a promising nanocarrier system for oral drug delivery with several advantages, including low toxicity, protection from degradation, high drug loading capacity for lipophilic drugs and scale-up capability [17–19]. Negatively charged SLNs are easily captured by inflamed intestinal tracts because of the impaired intestinal epidermal cells and positive charges of the inflamed tissues [20, 21], whereas the burst release of encapsulated drugs is the major obstacle for drug delivery to the lower gastrointestinal tract [17, 18]. Although several types of surface-modified SLNs have been developed for controlling drug release and preventing early drug release [22, 23], the assembly and disassembly of the surface layer have been shown to be mainly dependent on alterations in charge interactions in response to changes in environmental pH [23]. However, the pH variation between the small intestine and large intestine is limited. Thus, drug delivery to inflamed colon segments remains a major challenge. In addition, stably coating a stimuli-responsive outer layer onto the hydrophobic inner core of SLNs is another major challenge.

**Fig. 1:**
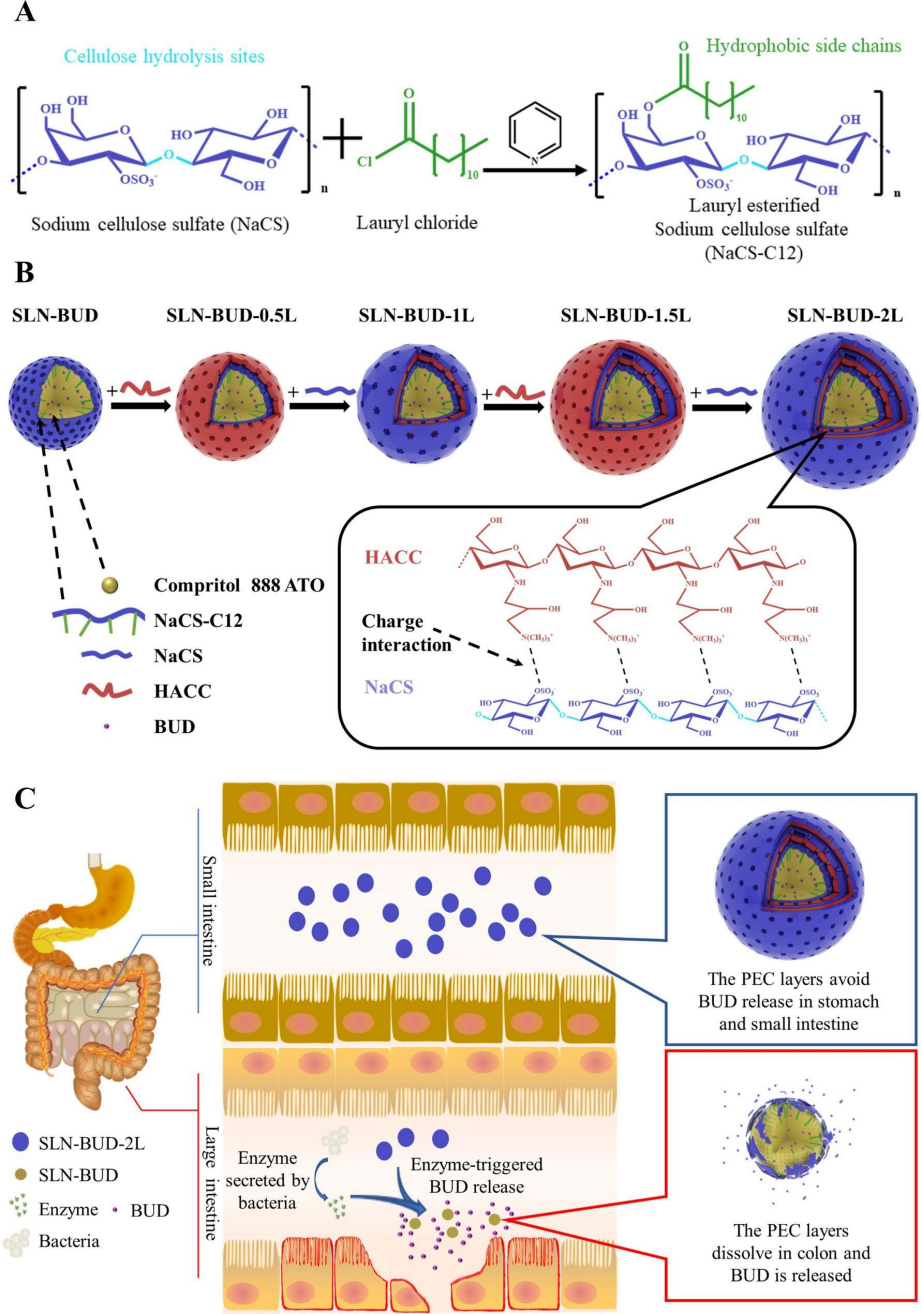
Solid lipid nanoparticle (SLN) synthesis procedure and GI flora-triggered drug topical release system. (**A**) Synthetic route of NaCS-C12. (**B**) The procedure of LBL self-assembly on the SLN template forming polyelectrolyte complex (PEC) layers. (**C**) The release mechanism of BUD from different generations of SLNs in different GI tracts

To address these problems, we designed and prepared colon-targeted SLNs encapsulating budesonide (BUD), which is a corticosteroid drug clinically approved for UC treatment, for oral administration. We synthesized a new negatively charged surfactant (NaCS-C12) by conjugating a hydrophobic carbon chain onto a NaCS polysaccharide backbone through ester linkages (Fig. 1A). The amphipathic NaCS-C12 not only stabilized the SLNs but also effectively anchored the responsive PEC layer onto the inner core via electrostatic forces and hydrophobic interactions. The PEC layer, which can be hydrolyzed by colon microflora-secreted cellulase, enabled the SLNs to release BUD in the colon. The negative charge and nanoscale particle size of the SLNs also enhanced BUD accumulation and maintenance in inflamed intestinal tissues. The particle size of the SLNs and the BUD release rate could be easily regulated by the number of coated layers. The 2-layer BUD-loaded SLNs (SLN-BUD-2L) with a proper particle size and stability showed an ideal colon-specific drug release profile in response to localized high cellulase activity. Importantly, in comparison with free BUD and nontargeted BUD-loaded SLNs, SLN-BUD-2L exhibited superior anti-inflammatory activity in a dextran sulfate sodium (DSS)-induced acute colitis mouse model, indicating the therapeutic potential of our colonic enzyme-responsive nanosystem for oral delivery.

## Materials and methods

### Materials

Chitosan quaternary ammonium salt (HACC) with an 95% degree of quaternization, and budesonide (BUD, 98%) were purchased from Macklin (Shanghai, China). Sodium cellulose sulfate (NaCS) was provided by Institute of Bioengineering, Zhejiang University. Cellulase was ordered from Meilunbio (Dalian, China). Lauroyl chloride, pyridine, and iron oleate were obtained from Aladdin (Shanghai, China). Compritol® 888 ATO was ordered from Gattefosse (Shanghai, China). Dextran sodium sulfate (DSS) was obtained from Yeasen (Shanghai, China). Pyrene was provided by Shanghai Dibo Chemicals Technology Co., Ltd. (Shanghai, China). Ethanol, acetaldehyde, sodium chloride, potassium chloride, dibasic sodium phosphate, and potassium dihydrogen phosphate were purchased from Sinopharm Chemical Reagent Co., Ltd (Shanghai, China). 3, 5-Dinitrosalicylic acid (DNS) reagent was obtained from Phygene Biotechnology Co., Ltd (Fuzhou, China).

### Synthesis of lauryl chloride-esterified NaCS (NaCS-C12)

The esterification of NaCS with long-chain fatty acids was performed according to the method previously reported for carboxymethyl cellulose esterification [24]. The reaction scheme is shown in Fig. 1A. In brief, NaCS (500 mg) was suspended in 20 mL of pyridine and stirred at room temperature, and lauryl chloride (0.4 mL) was added dropwise. Subsequently, the reaction was carried out at 80 °C for 3 h. Ethanol was added to the mixture to precipitate the crude product and the precipitate was obtained by vacuum filtration. Finally, the NaCS-C12 was obtained by precipitated (×3) in 3-fold excess ethanol and dried under vacuum.

### Characterization of NaCS-C12

Fourier transform infrared spectroscopy (FT-IR) analysis was performed with a Nicolet iS50 (Thermo Fisher, USA) spectrometer. Samples were pressed into KBr discs and measured at room temperature, and 32 scans with a resolution of 4 cm^− 1^ were measured in a wavenumber range of 400–4000 cm^− 1^. Proton nuclear magnetic resonance (^1^ H NMR) spectra were measured with an Avance III 500 MHz NMR spectrometer (Bruker, Switzerland) at 25 °C using DMSO-d6 as the solvent.

The critical micelle concentration (CMC) of amphiphilic NaCS-12 was measured by measuring the fluorescence spectra of pyrene [24]. In brief, a known amount of pyrene solution in benzene was added to a series of vials to give a final concentration of 6.0 × 10^− 5^ M. When benzene was evaporated completely, aqueous solutions of NaCS-C12 at various concentrations were added, sonicated in an ultrasonic water bath for 30 min, and stirred at room temperature overnight in dark. The fluorescence spectra of pyrene were measured with an F-4500 fluorescence spectrometer (Hitachi, Japan). The excitation wavelength was 340 nm, and the emission intensities measured at 373 nm (I_1_, of the first peak) and 383 nm (I_3_, of the third peak) were used to calculate the I_1_/I_3_ ratio. The CMC values were obtained based on the point of two tangents to the curve at the inflection.

### Preparation of PEC-coated and uncoated SLNs

SLNs were prepared according to a previously reported method [25] with some modifications. Briefly, Compritol® 888 ATO (500 mg) was melted in a glass vial at 85 °C. For the drug-loaded formulation (SLN-BUD), BUD (5 mg) was gradually added to the melted lipid with continuous stirring to ensure the dissolution of the drug. The aqueous phase was prepared by dissolving 100 mg of NaCS-C12 in 10 mL of distilled water. After warming up at the same temperature as the lipid phase, the aqueous solution was quickly added into the lipid phase, and then a Scientz-II D probe sonicator (Scientz, China) was utilized to sonicate the mixture for 20 min. During the ultrasound process, the coarse emulsion was constantly maintained at a temperature above 85 °C. Finally, the microemulsion was dispersed in 30 mL of prechilled water (4 °C) under stirring for 30 min to induce SLN formation.

The washless layer-by-layer (LBL) self-assembly of PEC layers on the SLN-BUD surface was carried out as shown in Fig. 1B [26, 27]. Before the LBL coating process, polymer solutions of anionic NaCS and cationic HACC were prepared at a concentration of 40 mg/mL and 10 mg/mL respectively. For the LBL buildup, SLN-BUD dispersion (1% w/v, 10 mL) was titrated with the cationic HACC aqueous solutions at different volume ratios of under gentle stirring for 30 min. To determine the minimum volume of HACC solution required to completely coat SLN-BUD nanocore surfaces, the HACC solution was step-wise added to the SLN-BUD dispersion and the mixture was stirred for at least 30 min. Then the zeta potential of the resulting nanoparticle was monitored, and the HACC solution was added until the results of the zeta potential reached stabilization. Subsequently, the second oppositely charged polyelectrolyte (NaCS) was added in a similar manner. The LBL assembly was carried out through the alternate addition of HACC and NaCS to form up to 3 bilayers. SLN-BUD with 1, 2, and 3 PEC bilayers were prepared for further studies.

### Physicochemical characterization of SLN-BUD with different numbers of PEC layers

The Z-average nanoparticle diameter (size), polydispersity index (PDI) and zeta potential surface charge of SLN-BUD with different numbers of PEC layers were assessed at 25 °C using the dynamic light scattering (DLS) method via a Zetasizer nano (ZS 90, Malvern Instruments, Malvern, UK).

Transmission electron microscopy (TEM, HT-7700, Hitachi, Japan) was used to investigate the shape and surface morphology of the nanoparticles.

The drug loading capacity (DL) and encapsulation efficiency (EE) of SLN-BUD were calculated by measuring the concentration of free BUD in the supernatant after ultrafiltration by 10 kDa filter tubes (Millipore, Germany). The free BUD in the aqueous solution was quantitatively detected with a UV‒Vis spectrophotometer at 245 nm. DL and EE were calculated according to the following equations:

EE% = [(M1-M2)]/M1] * 100.

DL% = [(M1-M2)/M3] * 100.

where M1 is the total BUD used in the formulation, M2 is the amount of BUD in the supernatant, and M3 is the amount of drug-loaded nanoparticles. The amount of drug-loaded nanoparticles was determined by directly weighing the freeze-drying drug-loaded nanoparticles.

The short-term stability of SLN-BUD-2L was investigated under different temperatures (4℃ and 25℃), and the particle size, PDI, zeta potential and EE of the SLNs were monitored using dynamic light scattering (Malvern Zetasizer, UK) and UV‒Vis spectroscopy at specified time intervals (0, 4, 7 days of storage).

### BUD release from SLN-BUD with different numbers of PEC layers

The BUD release profiles of SLN-BUD with different numbers of PEC layers (SLN-BUD, SLN-BUD-1L, SLN-BUD-2L, SLN-BUD-3L) were investigated using a dialysis method. Briefly, 3 mL of 1% w/v SLNs (BUD 0.167 mg/mL) with or without cellulase (1200 U/L) [28] was transferred into a dialysis bag with a molecular weight cutoff of 14,000 Da, and then a dialysis tube was immersed in 30 mL of PBS as the release medium. At selected time intervals, 0.5 mL release medium was collected, and 0.5 mL fresh PBS solution was used to replenish the solution. The BUD concentration was analyzed by UV‒Vis spectroscopy at 245 nm. The cumulative release was calculated as the total percentage of drug released through the dialysis membrane over time. Experiments were run in triplicate (n = 3). The data shown in the graphs represent the averages ± standard deviations (SDs).

### ***In vitro*** drug release of SLN-BUD-2L

The *in vitro* drug release profile of SLN-BUD-2L was investigated in three different simulation fluids, including simulated gastric fluid (SGF, pH 1.2), simulated small intestinal fluid (SIF, pH 6.8) and simulated colonic fluid (SCF, pH 7.4, containing 1200 U/L cellulase). For SCF, since the enzyme could not penetrate the dialysis bag, cellulase was added to the SLN-BUD-2L dispersion instead of the release medium to simulate colonic conditions. Three milliliters of SLN-BUD-2L (1% w/v) containing 0.5 mg of BUD was placed in a dialysis bag (molecular weight cutoff of 8000–14,000 Da), and the bag was placed in one of the three simulation fluids. Then, the whole setup was placed in an incubation shaker (100 rpm) at 37 °C. At selected time points, 0.5 mL simulation fluid was collected from the release medium, and the same volume of fresh release buffer was replaced. The released BUD was spectrophotometrically quantified by UV‒Vis at 245 nm.

The BUD release profile of SLN-BUD-2L was also investigated following the sequential immersion of samples in SGF, SIF and SCF to mimic the *in vivo* environmental changes in the GI tract after oral dosing. Three milliliters of SLN-BUD-2L (1% w/v) containing 0.5 mg of BUD was placed in a dialysis bag (molecular weight cutoff of 14,000 Da) and incubated in 30 mL of SGF (pH 1.2) for 2 h at 37 °C. The dialysis tube was then transferred to 30 mL of SIF (pH 6.8) for 3 h and finally placed in 30 mL of SCF (pH 7.4, containing 1200 U/L cellulase) for up to 24 h [29]. Then, 0.5 mL release medium was collected at certain time points and replaced with the same volume of fresh release medium. The BUD concentration in the release medium was measured in the same manner as previously mentioned.

### Animal experiments

All animal experiments were conducted with the approval of the Zhejiang University Experimental Animal Welfare and Ethics Committee under Institutional Animal Care and Use Committee guidelines. Male C57BL/6J mice (6–8 weeks; body weight: 20–25 g) and male SD rats (6–8 weeks; body weight: 180–200 g) were obtained from the Animal Center of the Hangzhou Medical College. Animals were housed in standard mouse cages under standard conditions, with ad libitum access to water and food.

### Intestinal lavage fluid drug release of SLN-BUD-2L and cellulase activity detection

*Ex vivo* drug release was performed using intestinal lavage fluids from rats. Briefly, SD rats (180–200 g) were euthanized by cervical dislocation. Next, the large intestine and small intestine were excised, and the intestinal contents were individually collected, weighed and added at 15% w/v to phosphate buffer (pH 6.8). The PBS buffer containing intestinal content was stirred in a shaking incubator at 37 °C overnight and then centrifuged at 10,000 × g for 5 min to obtain small intestine lavage fluid (SILF) or large intestine lavage fluid (LILF). Similar *in vitro* drug release experiments were performed for SLN-BUD-2L in SILF or LILF.

The cellulolytic activity in SILF and LILF was quantified using 9 mL of NaCS solution (1% w/v) as a substrate, and 1 mL of LILF or SILF was added as the enzyme solution. The hydrolysis reaction was carried out at 37 °C and 200 rpm for 1 h, and a 1 mL sample was taken from the mixture. The concentration of reducing sugar in the sample was detected by the Ghose method, and a calibration plot was established over a range of glucose concentrations [30]. The cellulolytic activity unit was defined as the amount of enzyme required to catalyze the hydrolysis of NaCS to produce 1 µmol of reducing sugar per min at 37 °C and pH = 7.0.

### Anti-inflammation evaluation of SLN-BUD-2L

Thirty male C57BL/6J mice were randomly assigned to 5 groups (n = 6): group 1: healthy + PBS; group 2: model + PBS; group 3: model + free BUD; group 4: model + SLN-BUD; and group 5: model + SLN-BUD-2L. Except for the group of healthy mice, which were provided with pure water, the other mice were provided with drinking water containing 3% (w/v) DSS for 7 days to induce colitis, followed by normal water during the next 5 days of treatment. In a therapeutic setting, the mice in groups 3, 4, and 5 were orally administered free BUD, SLN-BUD, and SLN-BUD-2L, respectively. SLN formulations (0.15 mL) containing equivalent doses of BUD (0.168 mg/kg/day) were orally gavaged daily from day 8 to day 12 [23]. The mice in groups 1 and 2 were orally dosed with PBS. Changes in the body weight, fecal bleeding, and stool consistency of the mice were observed daily. Disease activity index (DAI) scores, which were defined as the summation of the stool consistency index (0–4), fecal bleeding index (0–4), and weight loss index (0–4), were evaluated (Fig. 5E) [31]. The fecal bleeding index was determined with a BO test kit (BASO, China).

**Fig. 2:**
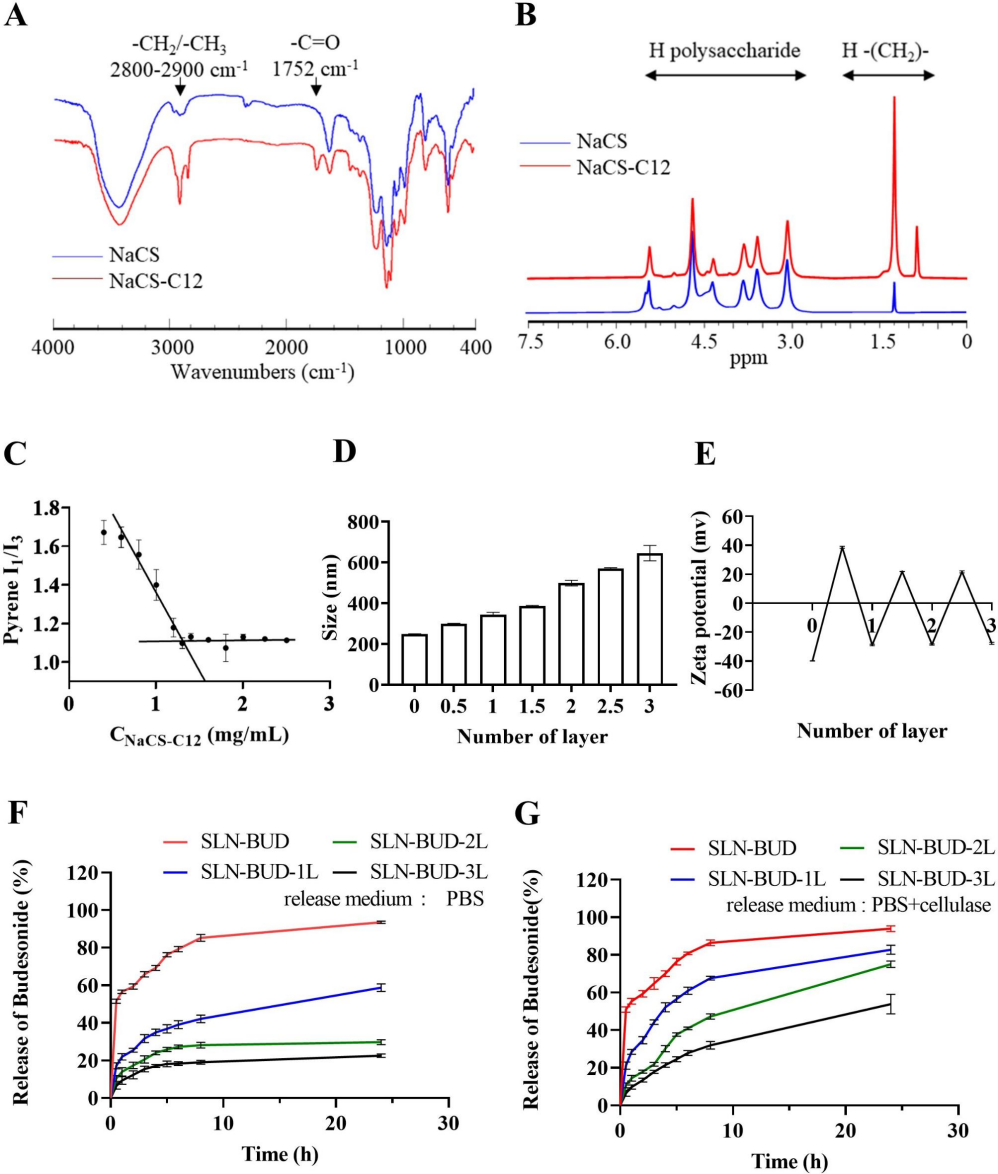
Characterization of NaCS-C12 and SLNs coated with different numbers of PEC layers. (**A**) FT-IR spectra of NaCS and NaCS-C12. (**B**) ^1^ H NMR spectra of NaCS and NaCS-C12. (**C**) CMC of NaCS-C12. (**D**) Z-average size of SLNs coated with different numbers of PEC layers and (**E**) the zeta potential of SLNs. (**F**) Cumulative BUD release profiles of SLNs coated with different numbers of PEC layers in PBS and (**G**) in PBS with cellulase (1200 U/L).

On the last day of the experiment, the mice were euthanized, and entire colons and spleens were collected. The lengths of the colons were measured and washed with saline, and the spleens were weighed. Then, 2 cm distal colon sections were used for histological assessment. The colon segments were fixed in 10% formalin solution for 48 h and then embedded in paraffin. Then, the colon samples were sectioned at a thickness of 5 μm and stained with hematoxylin and eosin (H&E) for histological analysis, followed by imaging with an optical microscope (Nikon Eclipes E200, Japan) equipped with a camera. The activity of myeloperoxidase (MPO) in the colon tissue was measured with an MPO test kit (Elabscience, China) following the operation manual. One unit of MPO activity was defined as the amount of MPO needed to degrade 1 µmol of peroxidase per minute. To determine the interleukin 6 (IL-6) and tumor necrosis factor alpha (TNF-ɑ) concentrations in the colon tissue, colon segments in 50 mM phosphate buffer (pH 6.0) were homogenized (1:10 w/v) at 4 °C and centrifuged for 10 min at 10,000 × g. The levels of cytokines in the resulting supernatants were determined with a commercial ELISA kit (Nan Jing Herb Source, China).

### Statistical analysis

Statistical analysis was performed using GraphPad Prism 9. Data are shown as the means with the SDs in parentheses. A t test was used to assess the significance of the difference between two means. The statistical significance of the differences was expressed as p values * < 0.05, ** < 0.01, *** < 0.001, **** < 0.0001.

## Results

### Synthesis and characterization of the negatively charged amphipathic surfactant NaCS-C12

To fabricate an effective and stable SLN surface coating, amphipathic NaCS-C12 was synthesized by grafting hydrophobic carbon chains onto the NaCS polysaccharide backbone through ester linkages (Fig. 1A). FT-IR spectra were collected for the structural characterization of NaCS and NaCS-C12 (Fig. 2A). The broad peak in the region of 3000–3700 cm^− 1^ was attributed to the stretching vibration of the − OH group. Compared to that in the NaCS spectrum, the intensity of the hydroxyl group in the NaCS-C12 spectrum was lower, indicating that − OH groups were substituted. This phenomenon was associated with an increase in the intensity of the characteristic signal of alkyl bonds at 2900-2800 cm^− 1^, corresponding to the presence of long fatty chains. The appearance of new bands at 1740 cm^− 1^ (C = O stretching) was also observed, indicating the vibration of ester carbonyl groups. These results suggested the successful esterification of cellulose with fatty acids. ^1^ H NMR spectroscopy was used to further confirm the presence of the fatty chains on the cellulose backbone. As shown in Fig. 2B, the peaks in the region of 3.0-5.5 ppm were indicative of the cellulose backbone protons, while the appearance of peaks from 0.5 to 1.5 ppm in the NaCS-C12 spectrum reflected the introduction of the alkyl chains. CMC is a crucial parameter for revealing the self-assembly behavior of NaCS-C12. A sharp change in the intensity ratio of the first and third vibronic emission bands of pyrene in the fluorescence measurements indicated the onset of micellization, causing a decrease in the polarity of the pyrene microenvironment [24]. Therefore, this change in the I_1_/I_3_ ratios as a function of surfactant concentration was used to determine the CMC (Fig. 2C). On the basis of the crossover point of the plotted curve, the CMC value (1.25 mg/mL) was determined.

### Optimization of PEC layer-coated SLNs

Alternating polyelectrolyte layers (NaCS and HACC layers) were self-assembled LBL on the SLN surface (Fig. 1B). DLS analysis indicated that this LBL assembly resulted in a significant increase in particle size (Fig. 2D). The particle sizes of SLN-BUD, SLN-BUD-1L, SLN-BUD-2L and SLN-BUD-3L were 248.5 nm, 385.9 nm, 498.6 and 645.2 nm, respectively. Despite the size increase, the PDI values were maintained at approximately 0.09∼0.21, suggesting that the nanoparticles were monodisperse. Meanwhile, the deposition process of alternating polyelectrolyte layers was accompanied by sequential charge reversal (Fig. 2E), indicating LBL self-assembly via electrostatic interactions. SLN-BUD displayed a surface charge of − 39.9 mv, owing to the presence of NaCS-C12 with a sulfate group on the nanoparticle surfaces. The zeta potential of SLN-BUD was converted to + 38.6 mv after HACC deposition. During the LBL coating process, NaCS and HACC layers were alternately coated on the nanoparticle surface, causing the zeta potential to switch between − 28.4 mv and + 21.8 mv.

An *in vitro* drug release study in the absence or presence of cellulase was conducted to demonstrate the enzyme responsiveness of SLNs with different numbers of PEC layers (SLN-BUD, SLN-BUD-1L, SLN-BUD-2L, and SLN-BUD-3L). As shown in Fig. 2F, the release of BUD from the uncoated SLN-BUD proceeded rapidly, with almost 56.6% of the loaded BUD released at 1 h. In contrast, by coating PEC layers on the SLN-BUD surface, the initial burst release of the BUD encapsulated in the SLNs was significantly reduced. The BUD release rates of SLN-BUD-1L, SLN-BUD-2L, and SLN-BUD-3L at 1 h were 21.9%, 13.9%, and 9.4%, respectively. At 24 h, the cumulative BUD release rates of SLN-BUD-1L, SLN-BUD-2L, and SLN-BUD-3L were 58.9%, 29.8% and 22.7%, whereas 93.6% of BUD was released from the uncoated SLN-BUD. Importantly, in the presence of cellulase, the cumulative BUD release rates (24 h) of SLN-BUD-1L, SLN-BUD-2L, and SLN-BUD-3L largely increased to 82.7%, 75.0%, and 53.8%, respectively (Fig. 2G). Cellulase is an enzyme capable of lysing the β-(1,4) glucoside linkages between the glucosidic units constituting cellulose chains. The cleavage of PEC layers by cellulase may have resulted in increased BUD release from the SLNs. Moreover, the enzyme-responsive release profile could be manipulated by adjusting the number of PEC layers deposited on the SLN surface. Considering its responsiveness and BUD release profile, SLN-BUD-2L was chosen for further studies.

### Physicochemical characterization of SLN-BUD and SLN-BUD-2L

**Fig. 3:**
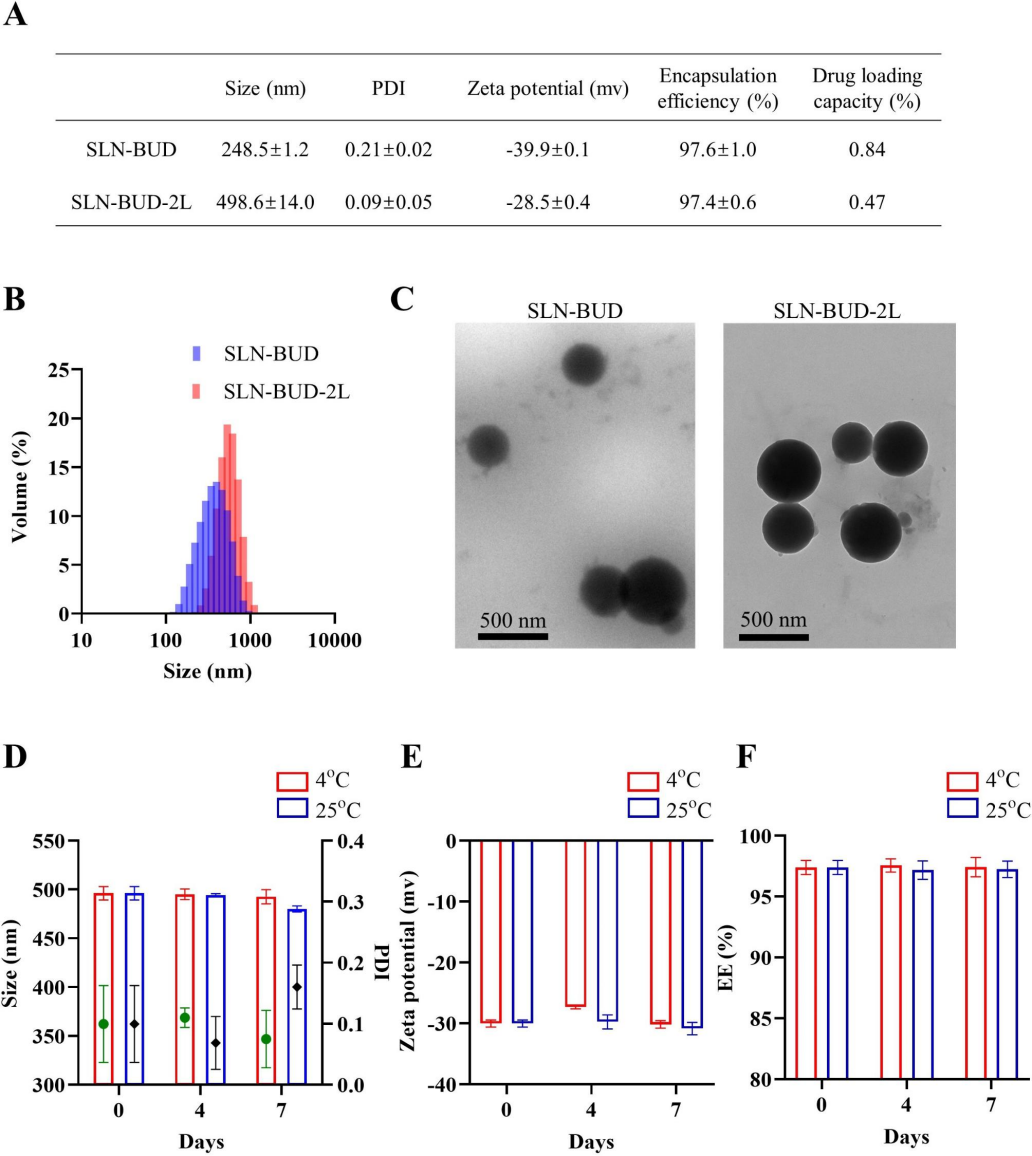
Characterization of SLN-BUD and SLN-BUD-2L. (**A**) Characterization of SLN-BUD and SLN-BUD-2L. (**B**) Size distribution analysis of SLN-BUD and SLN-BUD-2L by DLS. (**C**) TEM images of SLN-BUD and SLN-BUD-2L. (**D**) Size change, (**E**) zeta potential change, and (**F**) encapsulation efficiency of SLN-BUD-2L in the stability experiment at 4 and 25 °C

The particle size, PDI, zeta potential, EE, and DL of SLN-BUD and SLN-BUD-2L are shown in Fig. 3A, and the DLS curve is plotted in Fig. 3B. BUD (1% w/w of lipid mixture) was loaded into the SLNs during the SLN formation process. The hydrophobic properties of BUD caused a high partition between the lipid bilayer and aqueous phase, resulting in high BUD entrapment. During the coating process, the EE of BUD changed from 97.6% (SLN-BUD) to 97.4% (SLN-BUD-2L), and the DL changed from 0.84 to 0.47%. There were no remarkable changes in EE and DL between SLN-BUD and SLN-BUD-2L, which suggests that the drug entrapped in nanoparticles was retained during the LBL procedure. The TEM images of SLN-BUD and SLN-BUD-2L are presented in Fig. 3C, which indicated that the structural morphology of the SLNs was a nearly spherical shape in the dried state. It is important to note that electrostatic adsorption did not alter the shape of the template nanoparticles.

To evaluate the stability of SLN-BUD-2L, the changes in the particle size, PDI, zeta potential and EE of SLN-BUD-2L were measured during 7 days of storage at 4 and 25 °C (Fig. 3D-F**)**. No significant changes in the particle size and zeta potential were observed in the storage experiments, indicating the proper stability of SLN-BUD-2L.

### ***In vitro*** and ***ex vivo*** BUD release from SLN-BUD-2L

**Fig. 4:**
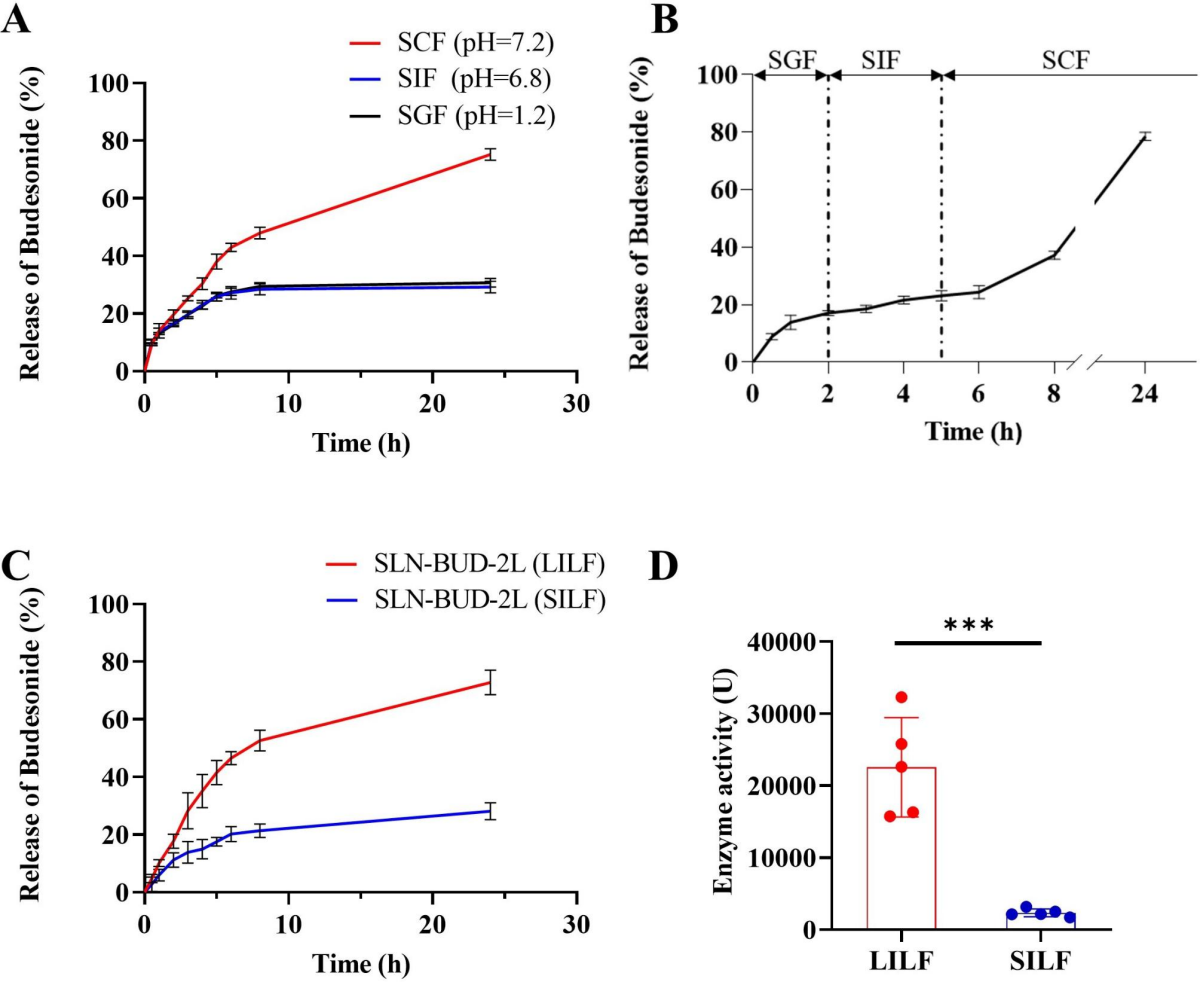
Drug release profiles of SLN-BUD-2L. (**A**) Cumulative BUD release curves of SLN-BUD-2L in SGF, SIF, and SCF. (**B**) Cumulative BUD release curves of SLN-BUD-2L in different simulated fluids over time. (**C**) Cumulative BUD release profiles of SLN-BUD-2L in LILF and SILF. (**D**) Cellulase activity in LILF and SILF.

To test whether SLN-BUD-2L could selectively release BUD in the colon microenvironment, we examined the drug release of SLN-BUD-2L in different media. First, the *in vitro* BUD release profile of SLN-BUD-2L was evaluated in SGF (pH = 1.2), SIF (pH = 6.8), and SCF (pH = 7.4, containing cellulase). As shown in Fig. 4A, **30**.7% and 29.2% of the encapsulated BUD was released from SLN-BUD-2L in SGF and SIF at 24 h, respectively. Notably, SLN-BUD-2L exhibited identical drug release profiles in SIF and SGF because the PEC layers consisting of HACC and NaCS did not respond to the pH value. As expected, the BUD release rate of SLN-BUD-2L was greatly increased in SCF, reaching 75.2% at 24 h. Next, BUD release was evaluated in different simulated fluids over time to simulate drug release in successive GI tract conditions (Fig. 4B). The residence time of SLN-BUD-2L in each medium was determined according to the transportation time of food in each GI tract segment [28]. In the first 2 h, 17.1% of BUD was released in SGF, and only another 6.0% was released over the next 3 h in SIF. However, the drug release of SLN-BUD-2L was accelerated in SCF (14.0% in 3 h), and approximately 78.5% of BUD was released in 24 h.

To provide further evidence that SLN-BUD-2L could selectively release BUD in the colonic environment, we measured the drug release profiles in LILF/SILF generated by SD rats (Fig. 4C). A similar BUD release rate was observed in SILF compared to that in SGF or SIF, whereas BUD was released from SLN-BUD-2L more quickly in LILF (72.8% at 24 h) than in SILF (28.2% at 24 h). We also tested the catalytic effect of the cellulase enzyme in LILF and SILF obtained from rats. (Fig. 4D). Relatively low enzyme activity (2349.2 U) was detected in SILF. In contrast, LILF showed an average enzyme activity of 22546.9 U, ranging from 32279.2 U to 15738.6 U. Taken together, the results of the drug release experiments and the enzyme activity measurements indicated that SLN-BUD-2L could deliver encapsulated BUD to the colon in response to the localized high cellulase activity.

### Therapeutic effect of SLN-BUD-2L in a DSS-induced colitis mouse model

**Fig. 5:**
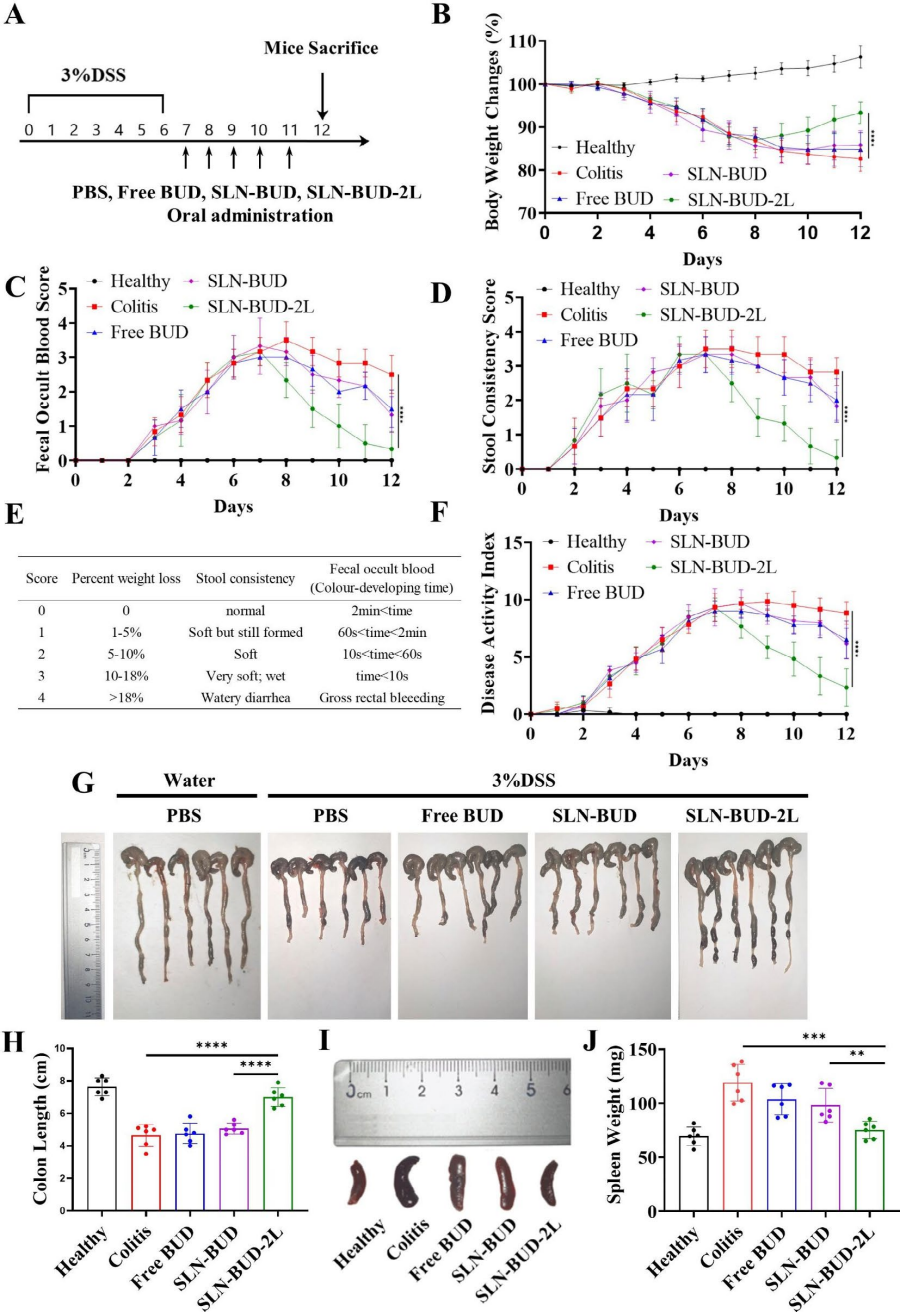
Therapeutic efficacy of SLN-BUD-2L in a DSS-induced mouse model. (**A**) Establishment of DSS-induced colitis in mice and treatment regimens. (**B**) Mouse body weight changes, (**C**) fecal occult blood score, and (**D**) stool consistency score changes of each group for 12 days. (**E**) Scoring system for calculating the DAI based on weight loss, stool consistency and the degree of intestinal bleeding. (**F**) DAI score changes in each group for 12 days. (**G**) Colon photographs and (**H**) the length of colonic tissues isolated from mice after 5 days of treatment. (**I**) Spleen photographs and (**J**) spleen weight for each group. All data are expressed as the means ± SDs (n = 6; *p < 0.05, **p < 0.01, ***p < 0.005, ****p < 0.001)

The *in vivo* therapeutic efficacy of SLN-BUD-2L against acute colitis was evaluated using a DSS-induced mouse model with the indicated treatment regimen (Fig. 5A). The development of colitis is characterized by body weight loss, fecal occult blood and stool consistency, the results of which are shown in Fig. 5B-D. The body weight of healthy mice slightly increased during the experiment, while the DSS-induced colitis mice showed reduced body weight. On day 12, the body weight loss of the colitis group, free BUD group and SLN-BUD group was 17.3%, 15.3% and 14.2%, respectively. However, a slight change in body weight (6.7%) was observed in the SLN-BUD-2L treatment group, indicating the great therapeutic efficacy of SLN-BUD-2L against acute colitis.

During the entire experiment, the fecal occult blood and stool consistency of each mouse were also monitored. The colitis group showed stool consistency and fecal occult blood scores of 2.8 and 2.5 points, respectively, on day 12. The free BUD and SLN-BUD groups showed moderately reduced scores, whereas the stool conditions of the SLN-BUD-2L group were largely recovered, and the point values of both scores decreased to 0.3 on day 12. In addition, the DAI calculated from the changes in body weight, stool consistency, and presence of hematochezia was also monitored to evaluate the severity of colitis (Fig. 5E-F). Consistent with the above observations, the DAIs of the free BUD and SLN-BUD groups slightly decreased on day 12 from 8.8 to 6.5 and 6.2, respectively. As expected, the DAI of the SLN-BUD-2L group significantly decreased to 2.3, suggesting the vigorous therapeutic potential of SLN-BUD-2L treatment against colitis.

All mice were sacrificed on day 12. Colon and spleen tissues were isolated and photographed (Fig. 5G-J). The length of the colon reflects the pathological degree of colitis because the scarring of the inflamed site could shorten the colon [32]. The healthy group exhibited an average colon length of 7.6 cm, while the colon length of the DSS-induced colitis groups dramatically decreased to 4.6 cm. The mean colon lengths of the free BUD group and the SLN-BUD group were 4.8 and 5.1 cm, respectively. Importantly, the colon length of the SLN-BUD-2L group was 7.0 cm, which was much closer to the baseline value of healthy mice, indicating that SLN-BUD-2L significantly ameliorated UC. Moreover, the average spleen weight of the colitis group increased to 119.1 mg, which was markedly higher than that of the healthy group (69.6 mg). The average spleen weight of the SLN-BUD-2L group largely decreased to 75.2 mg, whereas the spleen weight of the free BUD and SLN-BUD groups only slightly reduced to 103.7 and 98.2 mg, respectively.

**Fig. 6:**
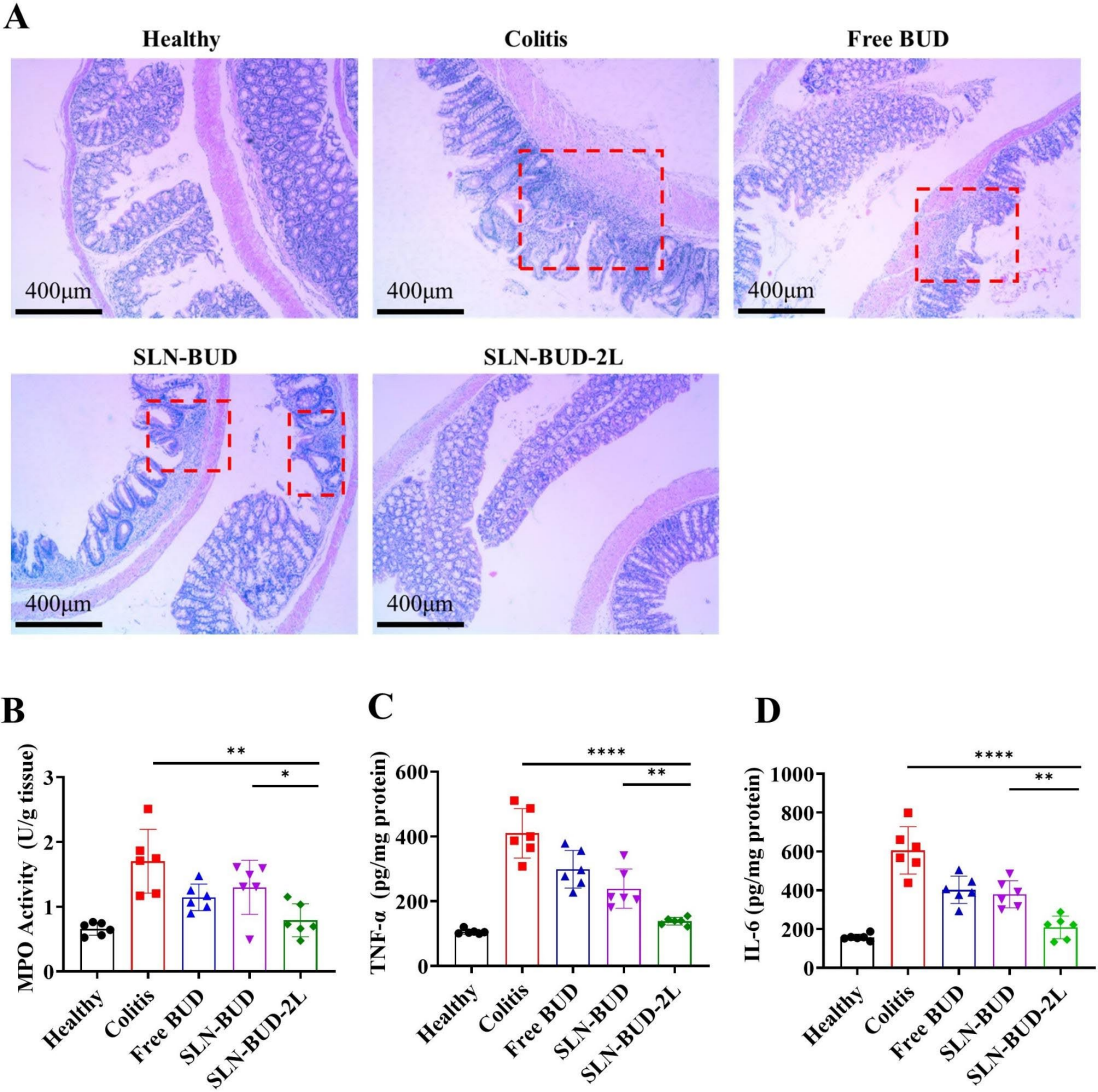
Histological evaluation of colon tissue and the expression levels of representative factors of inflammation in the colon for each group. (**A**) Histological evaluation of colon tissue in each group (the red box shows the irregular morphology of the colon). The tissue images are shown at 40 × magnification. Average MPO activity and proinflammatory cytokine expression in the healthy group, colitis group, free BUD-treated group, SLN-BUD-treated group and SLN-BUD-2L-treated group: MPO assay (**B**); TNF-α levels (**C**) and IL-6 levels (**D**). All data are expressed as the means ± SDs (n = 6; *p < 0.05, **p < 0.01, ***p < 0.005, ****p < 0.001)

The histological effects of free BUD, SLN-BUD and SLN-BUD-2L on DSS-induced colitis were examined by H&E staining, which directly reflected the degree of lesions in the colons. (Fig. 6A). The DSS colitis-positive control group exhibited obvious signs of inflammation, including irregular morphology of the colon (red box), edema and disruption of cryptal glands. Some of those signs of tissue damage were also observed in the free BUD and SLN-BUD groups. However, the DSS-induced mice treated with SLN-BUD-2L showed a nearly normal histological microstructure. We also detected the levels of inflammatory mediators, such as MPO, TNF-α and IL-6, on day 12. The colon tissue-associated MPO activity provides quantitative data on neutrophil infiltration that displays the severity of colitis. The MPO activity of the SLN-BUD-2L group was significantly reduced from 1.702 U/g tissue (colitis group) to 0.791 U/g tissue, exhibiting superiority over those of the free BUD (1.145 U/g tissue) and SLN-BUD (1.300 U/g tissue) groups (Fig. 6B). Similar results were also observed for TNF-α (Fig. 6C) and IL-6 (Fig. 6D).

## Discussion

BUD promotes wound healing and anti-inflammatory activity by targeting intracytoplasmic glucocorticoid receptors, resulting in the downregulation of several proinflammatory cytokines and subsequent inhibition of inflammatory cell proliferation and recruitment, but free BUD is rapidly absorbed in the proximal gastrointestinal tract and cleared through the liver [33]. BUD delivery to the site of inflammation in the colon is critical for UC therapy. SLNs have numerous advantages for oral drug delivery, such as organic solvent-free preparation, biocompatible and biodegradable ingredients, easy large-scale production and high loading capacity for lipophilic drugs [17, 34]. However, most orally administered medications are extensively absorbed in the upper part of the GI tract, resulting in low colonic local concentrations. The initial burst drug release of SLNs in the stomach [17, 18] and the degradation of SLN lipids by gastric lipases and pancreatic lipases limit the application of SLNs as colon-targeted drug carriers [35, 36].

In recent years, utilizing the pH gradient [23, 37] and transport time [38] in the GI tract to modulate particle degradation and drug release has gained much attention for colon-targeted drug delivery. The pH-sensitive oral formulations resist drug release in the acidic conditions of the stomach and small intestine, facilitating controlled drug release in a neutral pH environment. Naeem M et al. utilized Eudragit S100 and polyethyleneimine-coated lipid nanoparticles to achieve pH-sensitive colon-targeted BUD delivery. Their study demonstrated a higher drug release rate compared to that in our research [23]. However, preclinical and clinical evidence indicates that UC is associated with a local reduction in pH in the colon. The pH of the right/left colon in UC patients was reduced from 6.0 to 7.4 (normal colon) to 2.3–5.5 [39, 40], which may be attributed to malabsorption of the short-chain fatty acids, lactate and bicarbonate that are constantly generated by the microflora [40, 41]. Thus, pH-responsive formulations may not be suitable for delivering UC therapeutics. In addition, the time-controlled drug delivery strategy is also bottlenecked by the large variation in the rate of gastric emptying, small intestinal transport and colonic transit time in patients, resulting in unsatisfactory targeting specificity [40, 42]. To evaluate the colonic BUD delivery system, nanoparticles with a dual coating of time-sensitive (Eudragit FS30D) and pH-dependent (Eudragit RS100) polymers were used. The drug release curve of the time- and pH-sensitive nanoparticles was found to be similar to that of the SLN-BUD-2L release curve in this study, while the time-sensitive nanoparticles with excessively fast drug release did not exhibit colonic targeting [42].

Enzymes secreted by colon microflora can be used as specific stimuli to trigger colonic drug release (Fig. 1C). More importantly, the types and activities of bacterial glucosidases remain nearly unchanged in UC patients [43]. Thus, we utilized two cellulose-responsive natural polysaccharide derivatives, NaCS and HACC, as anionic and cationic counterparts to form PEC surface layers on SLNs [15, 44]. Compared to the widely used polysaccharide polyelectrolytes (chitosan and hyaluronic acid), NaCS and HACC have been regarded as strong polyelectrolytes because the electrostatic interaction between sulfate and quaternary ammonium occurs over a wide pH range. In addition, the high density of hydroxyl groups in NaCS/HACC could further enhance the stability and firmness of the PEC layer through hydrogen bond formation [45]. It is noteworthy that the initial coating of polyelectrolytes on the SLN surface is mainly dependent on electrostatic interactions [22, 46], which influence the stability of the core-shell structure. In this study, we synthesized amphipathic NaCS-C12 as a negatively charged surfactant to prepare SLNs, providing a strong negative zeta potential (-40 mV) to facilitate interactions with the cationic HACC layer. Importantly, the hydrophobic aliphatic alkane of NaCS-C12 could firmly insert into the lipid core of the SLNs, enabling stable surface modification for the grafting of PEC layers.

In addition to the colon-targeted controlled release capability, the negative charge and nanoscale particle size of SLN-BUD-2L could also contribute to its vigorous anti-UC activity by enhancing drug accumulation and retention in inflamed intestinal tissues. Compared to cationic nanoparticles, anionic nanoparticles may pass through the upper GI tract more easily, with reduced nonspecific binding to the small intestinal mucosa [47, 48]. Moreover, the accumulated eosinophils and neutrophils at the site of the ulcerated lesion secrete high concentrations of positively charged proteins, such as transferrin and eosinophil cationic protein (ECP) [40, 49], promoting the affinity for and adhesion to inflamed tissues of negatively charged nanoparticles. In addition, the disrupted intestinal barrier in the inflamed colon tissues could enhance the permeability of nanoparticles [50], while the enriched immune and inflammatory cells (macrophages, dendritic cells and neutrophils) are capable of internalizing and retaining these nanoparticles in the inflamed sites [51].

## Conclusions

In this study, cellulase-responsive SLNs were prepared for the colon-targeted delivery of BUD to treat UC for the first time. Amphiphilic NaCS-C12 was synthesized as a negatively charged surfactant, which acted as the surface anchor to facilitate the LBL assembly of the polysaccharide NaCS/HACC PEC layers on the SLNs. Compared to the uncoated SLN-BUD, SLN-BUD-2L with two PEC layers showed an ideal BUD release rate and cellulase-responsive drug release profile. The *in vivo* evaluation of its therapeutic effect in a DSS-induced colitis model revealed that orally administered SLN-BUD-2L exhibited superior anti-inflammatory efficacy over free BUD and uncoated SLN-BUD. Thus, cellulase-responsive SLN treatment could be a promising strategy for delivering localized UC therapeutics by the oral route.

## Data Availability

All data generated or analysed during this study are included in this published article.

## References

[1] Seyedian SS, Nokhostin F, Malamir MD (2019). A review of the diagnosis, prevention, and treatment methods of inflammatory bowel disease. J Med Life.

[2] Greenwood-Van B, Meerveld G, Pharmacology, editors.Springer International Publishing, Cham, Vol. 239, 2017.

[3] Cai Z, Wang S, Li J (2021). Treatment of inflammatory bowel disease: a Comprehensive Review. Front Med.

[4] Sairenji T, Collins KL, Evans DV (2017). An update on inflammatory bowel disease. Prim Care.

[5] Raine T, Bonovas S, Burisch J, Kucharzik T, Adamina M, Annese V (2022). ECCO Guidelines on therapeutics in Ulcerative Colitis: Medical Treatment. J Crohn’s and Colitis.

[6] Cunliffe RN, Scott BB (2002). Review article: monitoring for drug side-effects in inflammatory bowel disease.. Aliment Pharmacol Ther.

[7] Ashton JJ, Green Z, Kolimarala V, Beattie RM (2019). Inflammatory bowel disease: long-term therapeutic challenges. Expert Rev Gastroenterol Hepatol.

[8] Antonino RSCMQ, Nascimento TL, de Oliveira Junior ER, Souza LG, Batista AC, Lima EM (2019). Thermoreversible mucoadhesive polymer-drug dispersion for sustained local delivery of budesonide to treat inflammatory disorders of the GI tract. J Controlled Release.

[9] Date AA, Halpert G, Babu T, Ortiz J, Kanvinde P, Dimitrion P (2018). Mucus-penetrating budesonide nanosuspension enema for local treatment of inflammatory bowel disease. Biomaterials.

[10] Hanauer SB, Robinson M, Pruitt R, Lazenby AJ, Persson T, Nilsson LG (1998). Budesonide enema for the treatment of active, distal ulcerative colitis and proctitis: a dose-ranging study. Gastroenterology.

[11] Amidon S, Brown JE, Dave VS (2015). Colon-targeted oral drug Delivery Systems: Design Trends and Approaches. AAPS PharmSciTech.

[12] Eckburg PB, Bik EM, Bernstein CN, Purdom E, Dethlefsen L, Sargent M (2005). Diversity of the human intestinal Microbial Flora. Science.

[13] Froidurot A, Julliand V (2022). Cellulolytic bacteria in the large intestine of mammals. Gut Microbes.

[14] Wu Q-X, Guan Y-X, Yao S-J (2019). Sodium cellulose sulfate: a promising biomaterial used for microcarriers’ designing. Front Chem Sci Eng.

[15] Wang M-J, Xie Y-L, Zheng Q-D, Yao S-JA, Novel (2009). Potential Microflora-Activated carrier for a Colon-specific drug delivery system and its characteristics. Ind Eng Chem Res.

[16] Zhang Q, Lin D, Yao S (2015). Review on biomedical and bioengineering applications of cellulose sulfate. Carbohydr Polym.

[17] Mendoza-Muñoz N, Urbán-Morlán Z, Leyva-Gómez G, Zambrano-Zaragoza M, de la ¨Piñón-Segundo L, Quintanar-Guerrero E (2021). Solid lipid nanoparticles: an Approach to improve oral drug delivery. J Pharm Pharm Sci.

[18] Salah E, Abouelfetouh MM,  Pan  YH, Chen DM, XIe SY (2020). Solid lipid nanocarriers for enhanced oral absorption: A review. colloids and surfaces B: biointerfaces.

[19] Schwarz C, Mehnert W, Lucks JS, Müller RH (1994). Solid lipid nanoparticles (SLN) for controlled drug delivery. I. Production, characterization and sterilization. J Controlled Release.

[20] Collnot E-M, Ali H, Lehr C-M (2012). Nano- and microparticulate drug carriers for targeting of the inflamed intestinal mucosa. J Controlled Release.

[21] Hua S, Marks E, Schneider JJ, Keely S (2015). Advances in oral nano-delivery systems for colon targeted drug delivery in inflammatory bowel disease: selective targeting to diseased versus healthy tissue. Nanomedicine.

[22] Amasya G, Bakar-Ates F, Wintgens V, Amiel C (2021). Layer by layer assembly of core-corona structured solid lipid nanoparticles with β-cyclodextrin polymers. Int J Pharm.

[23] Naeem M, Oshi MA, Kim J, Lee J, Cao J, Nurhasni H (2018). pH-triggered surface charge-reversal nanoparticles alleviate experimental murine colitis via selective accumulation in inflamed colon regions. Nanomedicine.

[24] Li JF, Yang JS (2019). Synthesis of folate mediated carboxymethyl cellulose fatty acid ester and application in drug controlled release. Carbohydr Polym.

[25] Sakellari GI, Zafeiri I, Batchelor H, Spyropoulos F (2021). Formulation design, production and characterisation of solid lipid nanoparticles (SLN) and nanostructured lipid carriers (NLC) for the encapsulation of a model hydrophobic active. Food Hydrocoll Health.

[26] Bantchev G, Lu ZH, Lvov Y (2009). Layer-by-layer Nanoshell Assembly on Colloids through simplified Washless process. J Nanosci Nanotech.

[27] Santos AC, Sequeira JAD, Pereira I, Cabral C, Collado Gonzallez M, Fontes-Ribeiro C (2019). Sonication-assisted layer-by-layer self-assembly nanoparticles for resveratrol delivery. Mater Sci Eng C.

[28] Zhu L-Y, Lin D-Q, Yao S-J (2010). Biodegradation of polyelectrolyte complex films composed of chitosan and sodium cellulose sulfate as the controllable release carrier. Carbohydr Polym.

[29] Woraphatphadung T, Sajomsang W, Rojanarata T, Ngawhirunpat T, Tonglairoum P, Opanasopit P (2018). Development of Chitosan-Based pH-Sensitive polymeric Micelles containing curcumin for Colon-targeted drug delivery. AAPS PharmSciTech.

[30] Deshavath NN, Mukherjee G, Goud VV, Veeranki VD, Sastri CV (2020). Pitfalls in the 3, 5-dinitrosalicylic acid (DNS) assay for the reducing sugars: interference of furfural and 5-hydroxymethylfurfural. Macromol.

[31] Wirtz S, Popp V, Kindermann M, Gerlach K, Weigmann B, Fichtner-Feigl S (2017). Chemically induced mouse models of acute and chronic intestinal inflammation. Nat Protoc.

[32] Chung CH, Jung W, Keum H, Kim TW, Jon S (2020). Nanoparticles derived from the natural antioxidant Rosmarinic Acid ameliorate Acute Inflammatory Bowel Disease. ACS Nano.

[33] Abdalla MI, Herfarth H (2016). Budesonide for the treatment of ulcerative colitis. Expert Opin on Pharmacotherapy.

[34] Nasirizadeh S, Malaekeh-Nikouei B (2020). Solid lipid nanoparticles and nanostructured lipid carriers in oral cancer drug delivery. J Drug Delivery Sci Technol.

[35] Basha SK, Dhandayuthabani R, Muzammil MS, Kumari VS. Solid lipid nanoparticles for oral drug delivery. Materials Today: Proceedings. 2021;36:313–24.

[36] Yaghmur A, Mu H (2021). Recent advances in drug delivery applications of cubosomes, hexosomes, and solid lipid nanoparticles. Acta Pharm Sin B.

[37] Ali H, Weigmann B, Neurath MF, Collnot EM, Windbergs M, Lehr C-M (2014). Budesonide loaded nanoparticles with pH-sensitive coating for improved mucosal targeting in mouse models of inflammatory bowel diseases. J Controlled Release.

[38] Sangalli ME, Maroni A, Zema L, Busetti C, Giordano F, Gazzaniga A (2001). In vitro and in vivo evaluation of an oral system for time and/or site-specific drug delivery. J Controlled Release.

[39] Fallingborg J, Christensen LA, Jacobsen BA, Rasmussen SN (1993). Very low intraluminal colonic pH in patients with active ulcerative colitis. Digest Dis Sci.

[40] Cui MX, Zhang M, Liu K (2021). Colon-targeted drug delivery of polysaccharide-based nanocarriers for synergistic treatment of inflammatory bowel disease: a review. Carbohydr Polym.

[41] Tirosh B, Khatib N, Barenholz Y, Nissan A, Rubinstein A (2009). Transferrin as a Luminal Target for negatively charged Liposomes in the Inflamed Colonic Mucosa. Mol Pharm.

[42] Naeem M, Choi M, Cao J, Lee Y, Lkram M, Yoon S et al. Colon-targeted delivery of budesonide using dual pH- and time-dependent polymeric nanoparticles for colitis therapy. DDDT. 2015;3789. 10.2147/DDDT.S88672 PMC4516197 26229440

[43] Friend DR (2005). New oral delivery systems for treatment of inflammatory bowel disease. Adv Drug Deliv Rev.

[44] Andreica B-I, Cheng XJ, Marin L (2020). Quaternary ammonium salt of chitosan. A critical overview on the synthesis and properties generated by quaternization&nbsp;. European Polymer Journal.

[45] Borges J, Mano JF (2014). Molecular interactions driving the layer-by-Layer Assembly of Multilayers. Chem Rev.

[46] Finke JH, Schmolke H, Klages C-P, Müller-Goymann CC (2013). Controlling solid lipid nanoparticle adhesion by polyelectrolyte multilayer surface modifications. Int J Pharm.

[47] Lin C-H, Chen C-H, Lin Z-C, Fang J-Y (2017). Recent advances in oral delivery of drugs and bioactive natural products using solid lipid nanoparticles as the carriers. J Food Drug Anal.

[48] Kulkarni N, Jain P, Shindikar A, Suryawanshi P, Thorat N (2022). Advances in the colon-targeted chitosan based multiunit drug delivery systems for the treatment of inflammatory bowel disease. Carbohydr Polym.

[49] Peterson CGB, Carlson M (2002). A New Method for the quantification of Neutrophil and Eosinophil Cationic Proteins in feces: establishment of normal levels and clinical application in patients with inflammatory bowel disease. Am J Gastroenterol.

[50] Schmitz H, Barmeyer C, Fromm M, Runkel N, Foss H-D, Bentzel CJ (1999). Altered tight junction structure contributes to the impaired epithelial barrier function in ulcerative colitis. Gastroenterology.

[51] Lamprecht A (2010). Selective nanoparticle adhesion can enhance colitis therapy. Nat Rev Gastroenterol Hepatol.

